# A Facile Route to Synthesize Nanographene Reinforced PBO Composites Fiber via in Situ Polymerization

**DOI:** 10.3390/polym8070251

**Published:** 2016-07-04

**Authors:** Mingqiang Wang, Shuai Zhang, Jidong Dong, Yuanjun Song, Jiao Mao, Huaquan Xie, Yue Qian, Yudong Huang, Zaixing Jiang

**Affiliations:** 1 MIIT Key Laboratory of Critical Materials Technology for New Energy Conversion and Storage, State Key Laboratory of Urban Water Resource and Environment, School of Chemistry and Chemical Engineering, Harbin Institute of Technology, Harbin 150001, China; wangminqiang55@163.com (M.W.); dongjidong1122@126.com (J.D.); songyj@hit.edu.cn (Y.S.); 18746069239@163.com (J.M.); mr.scoke@foxmail.com (H.X.); qy1993@163.com (Y.Q.); 2 Interdisciplinary Nanoscience Center (iNANO), Aarhus University, DK-8000 Aarhus C, Denmark; inanoshuai@gmail.com

**Keywords:** nanographene, poly(*p*-phenylene benzobisoxazole) fiber (PBO fiber), polymer composites, conjugated polymer

## Abstract

The polymer matrix with introduced carbon-based nanofiber displays fascinating properties. They have inspired extensive research on the synthesis of polymer composites, which have been applied in catalysis, electronics, and energy storage. In this report, we reported a facile and efficient method to prepare poly(*p*-phenylene benzobisoxazole) (PBO)/nanographene (PNG) composites fibers via in-situ polymerization, accompanied by the reduction from (nanographene oxide) NGO to (nanographene) NG. By tuning the ratio of feeding PBO monomer to NGO, various composites fibers with 0.1–1 wt % contents of NG were obtained. The efficient PBO chains grafting made NG uniformly disperse in the PBO matrix, and it also increased the uniformity of the packing orientation of PBO chains. Consequently, the tensile strength, tensile modulus, and thermal stability of the obtained PNG composites fibers had been improved significantly. In addition, the composites fibers with 0.5 wt % NG exhibited a 25% increment in tensile strength, and a 41% enhancement in tensile modulus compared with neat PBO fibers. It reveals an excellent reinforcement to PBO composites fibers with NG.

## 1. Introduction

Graphene, as being a single-layer two-dimensional nano-carbon material, has been widely investigated since its first discovery in 2004 [1]. The excellent mechanical and thermal properties of graphene make it a promising candidate for practical applications in many fields, such as optics, catalysis, electronics, and composites materials [2,3,4,5]. Among these applications, the incorporation of graphene to polymers has offered significant potential for the development of advance composites materials [6,7,8,9]. However, both the experimental and theoretical studies have shown that the properties of graphene mainly depend on their size and geometric structures (the graphene can be divided into nanographene, graphene ribbon and graphene sheet according to their structures) [10]. Hence, precise control over graphene geometric structures is a key to explore their basic properties, and to further apply it in material science and industry.

Poly(*p*-phenylene benzobisoxazole) (PBO) fibers are considered to be one of the most promising high performance materials in spacecraft and aircraft industries, because of their excellent mechanical properties and outstanding thermal stability [11,12,13,14]. However, even for commercial PBO fibers, there is still a large gap between the actual tensile strength and the theoretical one. Improvements in fiber orientation and molecular-chain-packing order have been recommended as the main strategies to overcome this obstacle [15]. Carbon based materials, like graphene and carbon nanotube (CNT) are considered as the additives to achieve the goals [16,17,18]. Previously, some attempts have been made to fabricate PBO/grapheme [19,20] and PBO/CNT composites fibers [21,22,23,24]. It was found that the mechanical properties and thermal stability of PBO fibers were dramatically enhanced by the incorporation of a small amount of carbon nanofibller. Considering the different sizes between nanographene (NG) and graphene sheets, the PBO/nanographene (PNG) composites may exhibit different properties. However, like the difficulties encountered in graphene sheet, some unresolved and critical issues limit practical applications, such as, poor dispersion of nanofiller in polymer matrix, and weak interfacial interactions between the NG and the polymer matrix.

Nanographene oxide (NGO), which is similar to graphene oxide (GO), consists of a two-dimensional sheet with covalently bonded carbon atoms containing various oxygen functional groups, such as carbonyl groups on the basal planes and edges [25,26,27]. These oxygen-containing functional groups improve the compatibility and reinforcing efficiency of NGO in polymer matrix. Meanwhile, the functional groups on NGO make the uniform dispersion of NGO in the mixture composites possible, because the grafting points to PBO polymer offer strong interaction with PBO monomers. However, to the best of our knowledge, there is no report of the application of NGO nanofibller as a reinforcement material in polymer composites.

**Figure 1:**
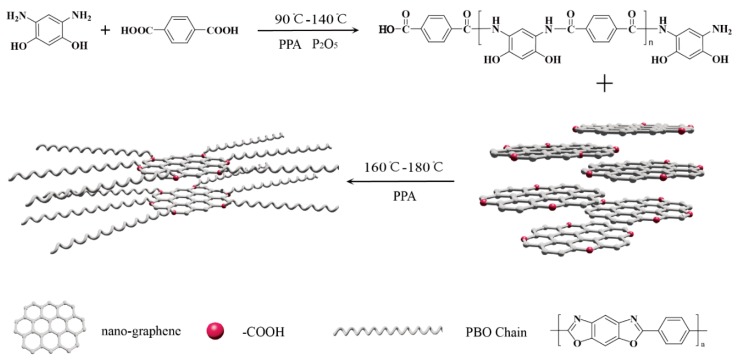
Scheme of the procedure for preparation of Poly(*p*-phenylene-benzobisoxazole) /nanographene (PNG) composites.

Herein, we reported a facile and efficient method to prepare PBO/nanographene (PNG) composites on the basis of carboxyl groups of NGO by in situ condensation polymerization with simultaneous reduction to NG from NGO, as shown in Figure 1. Modified NG was uniformly dispersed in composites. Moreover, we prepared continuous PNG composites fibers by a dry-jet wet-spinning process, and found that the tensile strength, tensile modulus, and thermal stability of PNG composites fibers had been improved significantly, compared to PBO, PBO/graphene and PBO/CNT composites. The composites fibers with 0.5 wt % NG exhibited a 25% increment in tensile strength, and a 41% enhancement in tensile modulus compared with neat PBO fibers, which open a promising application of PNG fibers in aircraft and other material engineering industries.

## 2. Materials and Methods

### 2.1. Materials

4,6-Diaminoresorcinol dihydrochloride (DAR·2HCl) was chemically synthesized according to the previous reports [28,29], Terephthalic acid (TPA) was purchased from Shanghai Reagents Company (Shanghai, China). Glucose, phosphoric acid (H_3_PO_4_), phosphorus pentoxide (P_2_O_5_), tin chloride dehydrate (SnCl_2_·2H_2_O) and other chemicals were purchased from Sigma-Aldrich (Shanghai, China).

### 2.2. The Synthesis and Purification of NGO

NGO was synthesized from glucose using a hydrothermal treatment method. The detailed process is as follows: 3.6 g glucose was dissolved in deionized water (40 mL) to form a clear solution, which was placed in a 100 mL teflon-lined autoclave chamber and kept at 180 °C for 4 h. After the reaction, the brown product was centrifuged at 10,000 rpm for 15 min to remove the black precipitates and the yellow supernatant was collected. The obtained yellow supernatant was further purified by dialyzing against DI water using a Spectra/Por dialysis membrane with molecular weight cut-off of 100 g/mol. The reason for the choice of using the dialysis membrane with low molecular weight cut-off is to avoid the loss of NGO during dialysis and remove the unreacted glucose. Then, the NGO obtained from yellow supernatant was freeze-dried.

### 2.3. Preparation of PNG Composites

A typical procedure for synthesizing PBO and PNG composites with different NGO contents of 0.1–1 wt % is depicted in Figure 1. First, 15 g new prepared DAR·2HCl, 51 g P_2_O_5_, 40 g H_3_PO_4_ and 1.5 g SnCl_2_·2H_2_O were added into a 500 mL three-neck round-bottom flask equipped with a nitrogen inlet/outlet and a mechanical stirrer. The reaction mixture was stirred until complete removal of hydrochloride at 90 °C under a nitrogen atmosphere and then 12 g TPA, 0.45 g NGO and another 25.6 g P_2_O_5_ were added to the mixture. After that, the reaction solution was stirred under nitrogen atmospheres at 120 °C for 4 h, 140 °C for 4 h, 160 °C for 4 h and 180 °C for 8 h to condense. Then, the PNG composites mixtures were obtained after the reaction. In the polymerization process, the concentration of PBO synthesized in phosphate (PPA) was controlled to be 12 wt %, the concentration of P_2_O_5_ at 83.5 wt % and the NGO content in the PNG composites was adjusted to be 0.1–1.0 wt %.

### 2.4. Fabrication of PNG Composites Fibers

The fresh synthesized PBO or PNG composites viscous solutions in PPA products were directly used as dopes for dry-jet wet-spinning. The dopes were first moved to the dope tank under the protection of nitrogen atmosphere and were heated at 180 °C for 5 h before spinning. The spinning parameters were as follows: Spinneret orifice diameter, 0.4 mm; air gap 30 mm; extruder pressure, 2.5 MPa; spinning velocity, 10 m/min; and spinning temperature, 180 °C. The drawn fibers were washed in running phosphoric acid (30%) solution, phosphoric acid (20%) solution, phosphoric acid (10%) solution and deionized water, respectively to wash PPA at room temperature and then dried in a vacuum at 60 °C for 24 h.

### 2.5. Characterization

The samples of NGO and PNG composites were characterized by Transmission electron microscopy (TEM: JEOL JEM-2010 operating at 200kV (Japan Electronics Co., Ltd., Tokyo, Japan), Fourier-transform infrared (FT-IR) spectrometer (Nicolet Nexus 670) (Nicolet Instrument. Inc., Madison, AL, USA), Wide angle X-ray diffraction (WAXD) patterns (D/Max-2550 PC rotating anode X-ray generator with Ni-filtered Cu Karadiation operated at 350 mA and 40 kV) (Rigaku Corporation, Tokyo, Japan), scanning electron microscopy (SEM: Netherlands Philips FEI Sirion) (Field Electron and Ion Co., Hillsboro, OR, USA), and thermogravimetry (TG) analyses (Netzsch STA449C from room temperature to 850 °C in air at a heating rate of 10 °C/min) (German NETZSCH Instruments Manufacturing Co., Ltd., Bavaria, Germany). The mechanical properties of PBO and PNG composites fibers were conducted using a staple fiber tensile tester (Universal Testing Machine-8402 Tester) (Shenzhen Sans Testing Machine Co. Ltd, Shenzhen, China) with a gauge length of 2 cm at a strain rate of 2% per minute The fibers’ diameters were measured with an optical microscope (equipped with CCD, CAMERA) (Canon, Tokyo, Japan). Fifty samples were tested in each case.

## 3. Result and Discussion

### 3.1. Synthesis of PBO/Nanographene Composites

The Figure 1 depicts the route to prepare PNG composites. With the temperature increasing gradually, TPA was initiated by DAR, and polymer chains of PBO step increased along with the consumption of monomers. Simultaneously, the carboxyl groups of NGO, which was reacted with amino groups at end of polymer chains, made the parts of the active polymer chains graft onto NGO, whose grafting mechanism was analogous to that of graphene-based PBO composites [19,20] or CNT-based PBO composites [21,22,23,24]. The viscosity of the mixture increased gradually with continuous development of polymer chains; and the PNG composites exhibited Weissenberg effect [30] at the end of reaction with the ratio of NGO lower than 1 wt %. This indicated PBO with high molecular weight were synthesized. The excessive carboxyl groups on NGO in the process of condensation polymerization disrupted the proportion between carboxyl groups and amino groups in the reaction system, which terminated the increasing of active chains. Hence, the higher feed ratio of NGO to PBO monomer meant the smaller length of grafted polymer chains, which corresponded to less section of grafted polymer.

### 3.2. Structure and Properties of NGO and PNG Composites

**Figure 2:**
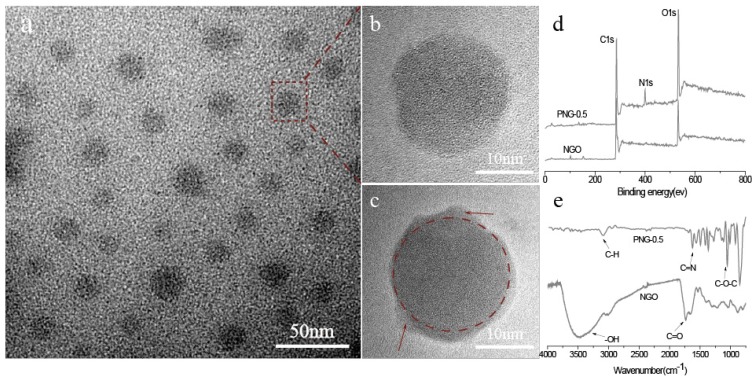
(**a**,**b**) TEM images of nanographene oxide (NGO) prepared from glucose; (**c**) the PNG composites (0.5%) were dissolved with methanesulfonic acid (MSA) in vacuum for 72 h; and (**d**) X-ray photoelectron spectroscopy (XPS) spectra of NGO and PNG-0.5; (**e**) Fourier-transform infrared (FT-IR) spectra of NGO and PNG-0.5.

Figure 2a shows the TEM morphology of NGO. The TEM (Figure 2a,b and Figure S1) observations show that NGO is uniform sheets with an average diameter of about 20 nm (Figure S2). Furthermore, the average height of these NGO is about 0.9 nm, as shown in Figure S3 and Figure S4. For nanoparticle-reinforced polymers, the state of nanoparticle in the composites is the key factor to decide the performance of composites. Thus, in this work, to confirm the existence of grafted PBO chains on NGO sheets, the PNG composites (0.5%) were dissolved with Methanesulfonic acid (MSA) in vacuum for 48 h. As PBO dissolve in MSA, we speculate that the residual solids should be NGO, because NGO cannot dissolve in MSA. Compared to NGO, the residual solids exhibit an encapsulating morphology, as marked by the arrows in Figure 2c, totally different from that of the prepared NGO. As the PNG composites were dissolved in MSA for 48 h, any free PBO chains should be dissolved and removed. Hence, the substance adhering NGO is deduced to be the grafted PBO chains, suggesting that the PBO chains were attached to the NGO surface via nucleophilic attack by amino groups on the carboxyl groups. In order to further characterize the residual solids, XPS, FT-IR and Roman spectrum were employed to analyze the composition, as shown in Figure 2d,e. In the XPS spectrum of OO (Figure 2d), two obvious peaks at 288.0 eV and 534.0 eV were observed, corresponding to C1s and O1s, respectively. No peak of nitrogen element was found. However, for the sample PNG-0.5, strong signal of N1s was detected at 402.0 eV besides the signals of C1s and O1s. Furthermore, for the sample PNG-0.5, the content of C and N increased corresponding to the decreased O when compared with NGO (as shown in Table S1), indicating that the plentiful of nitrogen element in PBO chains were grafted on NGO [24]. In the other hand, the FT-IR spectrum was used to detect characteristic bands of PNG-0.5% (Figure 2e). Compared with NGO, the new emerging broad band at 1625 cm^−1^ corresponds to the stretching vibration of the C=N groups in benzoxazole moiety. In addition, the O–C=O stretching vibration in carbonyl groups of NGO is not detected in the region of 1720 cm^−1^. This result proves that the benzoxazole ring is formed by cyclization of NGO and the PBO chains, and the obtained PNG composites are fabricated based on the covalent bonding interactions. Furthermore, the disorder mode (D band) at 1360 cm^−1^, and a tangential mode (G band) at 1580 cm^−1^, characteristic of NGO, are observed in all spectra (Figure S5). This implies that the graphite structure of NGO largely remains after the copolymerization. Because of the strong fluorescence base line, it is difficult to observe the change of D/G ratio. However, the high integration ratio of D and G bands suggests NGO have a high degree of copolymerization. All these results indicate the PBO chains grafted on NGO successfully.

To speculate the length of grafted PBO chains on NGO, we investigated the molecular weight of PNG composites. The weight-average molecular weight was calculated from Mark–Houwink equation, [η]dLg^−1^ = 2.77 × 10^−7^ × *M*_w_^1.8^ and the inherent viscosities of PBO and PNG composites were measured in MSA solution in an ubbelohde viscometer. The result showed that with increased NGO content, the weight-average molecular weights of PNG samples decreased, from 29,825 g/mol for pristine PBO to 28,412, 27,216, 26,487 and 23,012 g/mol for NGO-0.1, -0.2, -0.5 and -1 composites, respectively. This might be attributed to the excessive carboxyl groups on NGO, which change the ratio between amino groups and carboxyl groups in reaction system. Thus, higher ratios of NGO to PBO monomer lead to smaller lengths of grafted polymer chains on NGO, corresponding to the lower molecular weight section of PNG composites. Similar conclusions were acquired during in situ condensation polymerization of graphene-based PBO [19,20] composites and CNT-based PBO composites [23,24].

### 3.3. Properties of PNG Composites Fibers

**Figure 3:**
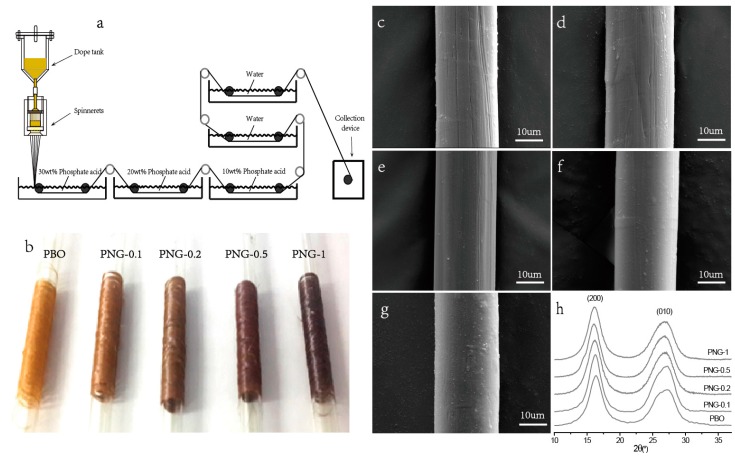
(**a**) Fabrication of Poly(p-phenylene-benzobisoxazole)(PBO) and PNG composite fibers using dry-jet wet-spinning technique; (**b**) Photograph of PBO and PNG composite continuous fibers collected using spools; (**c**–**g**) Representative scanning electron microscopy (SEM) images of PBO, PNG-0.1, PNG-0.2, PNG-0.5 and PNG-1 fiber, respectively; (**h**) Wide-angle X-ray diffraction patterns of PBO and PNG composites fibers.

According to previous literature, Graphene sheets or CNT are very effective to enhance the mechanical properties of polymer composites. Hence, to evaluate the enforcement effect of NGO, we synthesized a range of PNG composites fibers by a dry-jet wet-spinning process, of which the apparatus is illustrated in Figure 3a. The digital photos of the as-spun pristine PBO and PNG composites fibers (compositions of 0.1, 0.2, 0.5, and 1.0 wt % NGO, named PNG-0.1, PNG-0.2 PNG-0.5, and PNG-1, respectively) are shown in Figure 3b. It was observed that all the fibers had smooth surface. However, the color of the pristine PBO fibers was typically a bright-golden color, whereas PNG composites fibers were brown. This was attributed to the existence of NGO dispersed in the PBO fibers. In order to further learn the morphology of the PBO and the PNG fiber, SEM images are presented in Figure 3c–g. The images show that the diameters of the fibers are about 20 µm. There are slight cracks and micro fibrils on the PBO and the PNG fibers surfaces (Figure 3c). However, the surfaces of the PNG fibers are smoother than that of PBO fibers (Figure 3d–f) with the increased incorporation of NGO. It is suggested that NGO may prevent the polymer chains from movement, and rearrange them with the external force. That indicates the composites fibers are more compact than the PBO fibers. These effects may result in the increase of the mechanical and thermal properties of PNG fibers.

Moreover, Figure 3h demonstrates the change in orientation and crystal size of PBO fibers and PNG composites fibers with the rise of NGO content studied by WAXD patterns. For PNG composites fibers, the result is similar to that of PBO fibers and the diffraction peaks for NGO are not detected in the image. This means that NGO is well dispersed in the PBO fiber, and the crystal structure of PBO fibers remains after adding NGO. In the images, the two diffraction peaks at 2θ = 16.1° and 26.5° stand for “side-by-side” distance on (200) plane and “face-to-face” distance on (010) plane between two neighboring polymer chains, respectively. Furthermore, crystal size (*L*_hkl_) of fibers could be calculated using Scherrer’s equation.

(1) $$L_{\text{hkl}}=\frac{K\lambda }{B\cos\theta_{\text{hkl}}}$$

where λ is the wavelength of the Cu Ka beam (0.154 nm), *K* is a constant of 0.89, and B is the half-width of the (hkl) reflection at a scattering angle 2*θ*_hkl_. It can be found that NG is helpful for the packing of PBO molecular chains, which made fiber more compact. This result is similar to the work of GO-PBO composites [19,20] and CNT-PBO composites [21,22,23,24], in which NGO can act as a template for PBO orientation and a nucleating agent for PBO crystallization. The high aspect ratios of NGO make them easily to orient via a fiber spinning process. However, when the NGO content increased to 1 wt %, the orientation degree of PBO fiber decreased (Figure 3h). This might be attributed to that NGO incorporated with PBO backbone may affect the polymerization of PBO fibers, and the excessive carboxyl groups on NGO change the ratio between carboxyl groups and amino groups in reaction system. Therefore, the proper content of NGO with carboxyl groups facilitates the formation of interfaces with strong and flexible between NGO and PBO, increasing the polymer-reinforcing efficiency of NG.

### 3.4. Mechanical Properties

**Figure 4:**
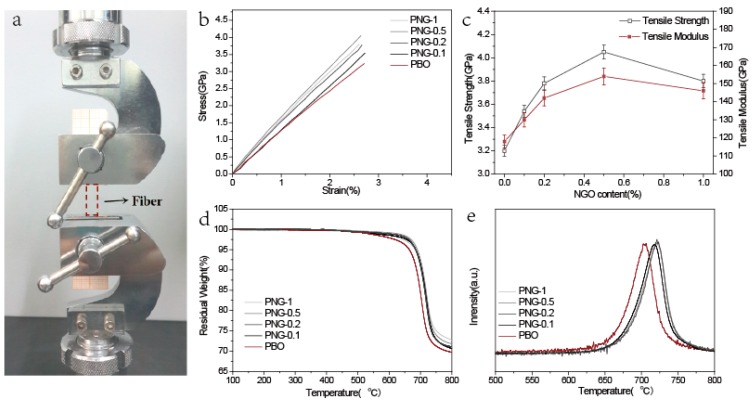
(**a**) Testing facility for mechanical properties (**b**) Typical stress–strain curves of PBO and PNG composites fiber with different NGO contents; (**c**) Tensile strength and tensile modulus change with increasing NGO content; (**d**) Thermal resistance of PBO and PNG composites fiber with different NGO contents: (**e**) Thermogravimetry (TG) curves and Differential Thermogravimetry (DTG) curves.

Mechanical properties are one group of the important properties of PBO or PNG composites fibers, because they are potential high-performance-engineering fibers. The testing apparatus is shown in Figure 4a and the mechanical properties of PNG composites fibers are shown in Figure 4b,c and Table 1. Figure 4b shows the typical stress–strain curves of PBO and PNG composites fiber, which show that the PNG composites fibers exhibit superior mechanical properties, compared to PBO fiber. In Figure 4c, pristine PBO fiber has a tensile strength of 3.2 GPa and tensile modulus of 118 GPa. The fiber with 0.1 wt % NGO has a tensile strength of 3.54 GPa, which is higher than that of pristine PBO fiber, and also exhibits a higher tensile modulus of 130 GPa. When the content of NGO increases up to 0.5 wt%, the tensile strength is significantly improved to 4.05 GPa accompanying further increasing of tensile modulus to 154 GPa. Moreover, by increasing the ratio of NGO, the break elongation decreases from 2.83% for pristine PBO to 2.68% for NG-0.5 composites fibers (Figure 4b). Undoubtedly, the excellent performance could be attributed to the good dispersion of NGO into the PNG fibers and strong interfacial interaction restricted the movement of the PBO chains when applied the force, which increased the tensile strength and tensile module, and decreased the elongation at break of the PNG composites fibers. However, the tensile strength of PNG composites fiber starts to decrease when the content of NGO increased to 1.0 wt %, and the tensile modulus of the PNG composites fibers had a similar tendency. The decrease of the tensile properties at higher content of NGO loading (1 wt %) might be ascribed to the aggregation of NGO due to the strong Van der Waals forces. The NGO agglomerates would form obstacles, causing bad interaction with the PBO chains and limiting energy rapid dissipation during the fracture process, which was consistent with XRD results. In turn, good dispersion of NGO in PNG composites fibers could help to dissipate the energy in the process of fracture.

The outstanding tensile properties of PNG composites fibers with different content of NGO suggest that the large ratios of NGO act as a platform for the condensation polymerization to obtain an outstanding dispersion of NGO within the PNG fibers. It results in strong interfacial interaction caused by the chemical bonding between NGO and the polymer chains. Moreover, optimized content of NGO improved the efficiency to increase tensile properties of PNG composite fibers. Hence, the method of polymer chains grafting modulated a flexible and hierarchical interphase between the NGO and polymer chains, which provide an effective method to transfer the load.

### 3.5. Thermal Properties

The thermal behaviors of pristine PBO and PNG with different NGO contents of 0.1–1.0 wt % composites fibers are studied by TG and DTG (Figure 4d,e). The experiments were conducted in nitrogen atmosphere at a heating rate of 10 °C /min. The presence of NGO observably enhances the thermal stability of the PNG composites fibers, because both the maximum mass loss temperature and onset decomposition temperature are shifted toward higher temperatures and the decomposition rate becomes slower, as shown in Figure 4d,e and Table 1. The onset decomposition temperature for PBO in air increases from 633 °C to 643 °C, 646 °C, 652 °C and 644 °C for PNG-0.1 PNG-0.2, PNG-0.5, and PNG-1, respectively. The residual carbon contents of PNG composites fibers are much higher than that of PBO fiber at 800 °C. The improvement of the thermal stability of PBO fiber resulted from the formation of crosslinking network structures by NGO and PBO oxazole rings. Hence, the carbon surface of NG in the PNG fibers might act as a platform to enhance the char formation degree of PBO, and that improves the thermal stability of PBO fiber. Moreover, the increased thermal stability of PNG fibers may be ascribed to the higher thermal conductivity of NGO, which may promote heat dissipation within the PNG composites fibers. Therefore, it can be concluded that the covalent bonding at the PNG composites interface is expected to improve their thermal stability.

## 4. Conclusions

In this work, we developed a novel facile route to prepare PBO/nanographene (PNG) composites via in situ polymerization. The chains of PBO were effectively grafted onto NGO through the reaction between the carboxylic groups on NGO, and the terminal amino ends of PBO chains accompanied by reducing NGO to NG. The grafted NG showed a good dispersion in the polymer, which provides great reinforcement to the PNG composites fibers. The mechanical properties and thermal stability of PNG composites fibers were greatly improved, even with low NGO content: A approximately 41% increase in tensile modulus and 25% improvement in tensile strength compared with PBO fibers. The mechanical properties of PNG composites fibers were also better than reported CNT-PBO and GO-PBO composites fibers [16,17,18,19,20,21]. Hence, this in situ polymerization opens a new avenue to fabricate nanographene-based composites, which will inspire the further developments and applications of PBO composites and other polymers.

## Figures and Tables

**Table 1:** Mechanical and thermal properties of PBO and PNG composites fibers.

Sample	Fiber diameter (μm)	Tensile modulus (GPa)	Strain to failure (%)	Tensile strength (GPa)	Decomposition temperature (°C)
PBO	20 ± 3	113 ± 6.6	2.83 ± 0.03	3.20 ± 0.32	633
PNG (0.1 wt %)	20 ± 2	128 ± 5.2	2.77 ± 0.03	3.54 ± 0.26	643
PNG (0.25 wt %)	20 ± 1	137 ± 4.3	2.74 ± 0.02	3.78 ± 0.18	646
PNG (0.5 wt %)	20 ± 2	151 ± 6.2	2.68 ± 0.02	4.05 ± 0.33	652
PNG (1 wt %)	20 ± 2	144 ± 5.6	2.63 ± 0.03	3.8 ± 0.19	644

## Supplementary Materials

Supplementary Materials can be found at www.mdpi.com/2073-4360/8/7/251/s1.
