# Supplementary material for: A Facile Route to Synthesize Nanographene Reinforced PBO Composites Fiber via in Situ Polymerization
Source: Polymers (Basel). 2016 Jul 4;8(7):251. doi: 10.3390/polym8070251 (PMC6432332; doi:10.3390/polym8070251)
Supplement: Supplementary file 1 [file polymers-08-00251-s001.pdf]

Supplementary Materials: A Facile Route to Synthesize Nanographene Reinforced PBO Composites Fiber via in Situ Polymerization

Mingqiang Wang, Shuai Zhang, Jidong Dong, Yuanjun Song, Jiao Mao, Huaquan Xie, Yue Qian, Yudong Huang and Zaixing Jiang

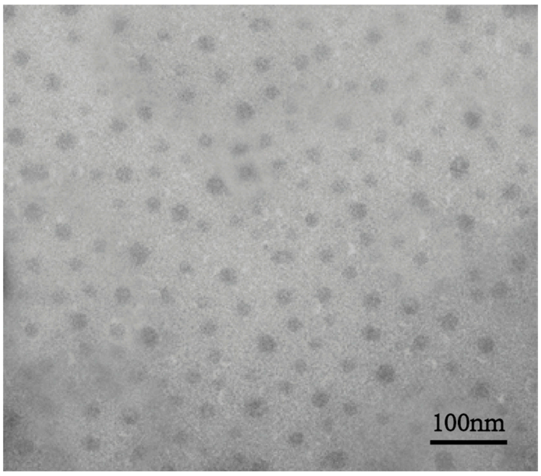

Figure S1. TEM images of NGO prepared from glucose.

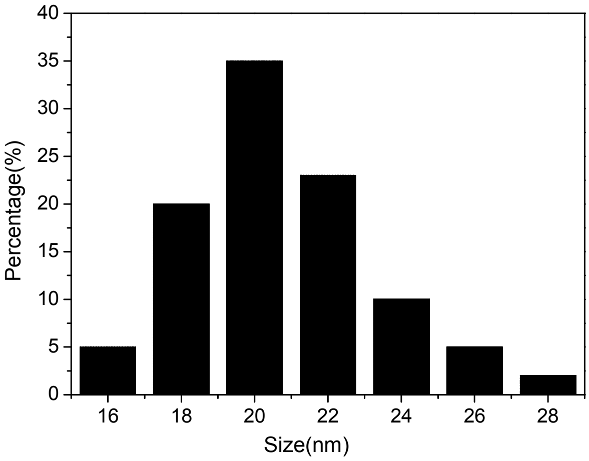

Figure S2. The size distribution of NGO.

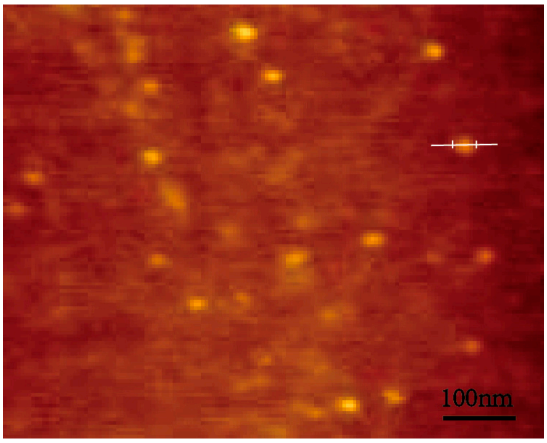

Figure S3. AFM image of the NGO deposited on freshly cleaved mica substrates.

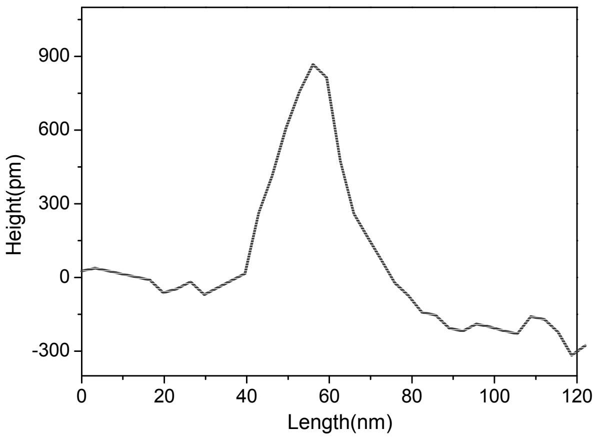

Figure S4. Height profile along the line in Figure S3.

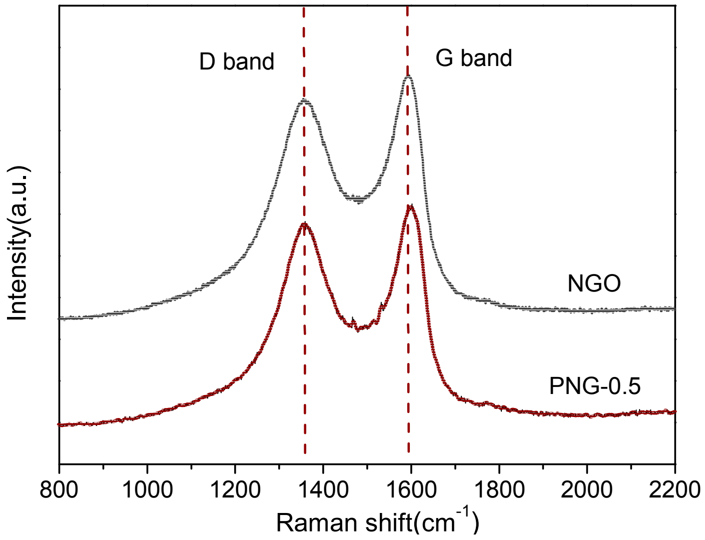

Figure S5. Raman spectra of NGO and PNG-0.5.

Table S1. The element content of NGO and residual solids.

Content	C (%)	O (%)	N (%)
NGO	69	31	0
Residual solids	80.07	16.24	3.69

© 2016 by the authors; licensee MDPI, Basel, Switzerland. This article is an open access article distributed under the terms and conditions of the Creative Commons Attribution (CC-BY) license (http://creativecommons.org/licenses/by/4.0/).
